# Transcriptomic data of *Arabidopsis* hypocotyl overexpressing a heterologous *CsEXPA1* gene

**DOI:** 10.1016/j.dib.2017.09.050

**Published:** 2017-09-22

**Authors:** Iqmal Asyraf Ilias, Othman Babul Airianah, Syarul Nataqain Baharum, Hoe-Han Goh

**Affiliations:** a Institute of Systems Biology, Universiti Kebangsaan Malaysia, UKM, Bangi 43600, Selangor Darul Ehsan, Malaysia; b Faculty of Science and Technology, Universiti Kebangsaan Malaysia, UKM, Bangi 43600, Selangor Darul Ehsan, Malaysia

**Keywords:** 3′ mRNA sequencing, Cell wall, Expansin, Hypocotyl, QuantSeq

## Abstract

Expansin increases cell wall extensibility to allow cell wall loosening and cell expansion even in the absence of hydrolytic activity. Previous studies showed that excessive overexpression of expansin gene resulted in defective growth (Goh et al., 2014; Rochange et al., 2001) [1,2] and altered cell wall chemical composition (Zenoni et al., 2011) [3]. However, the molecular mechanism on how the overexpression of non-enzymatic cell wall protein expansin can result in widespread effects on plant cell wall and organ growth remains unclear. We acquired transcriptomic data on previously reported transgenic Arabidopsis line (Goh et al., 2014) [1] to investigate the effects of overexpressing a heterologus cucumber expansin gene (*CsEXPA1*) on the global gene expression pattern during early and late phases of etiolated hypocotyl growth.

**Specification Table**

**Table:** 

Subject area	Biology
More specific subject area	Molecular Biology of Plant Development
Type of data	3′ mRNA Sequencing Reads
How data was acquired	Sequencing data were acquired through Illumina HiSeq. 2500 sequencing of QuantSeq libraries prepared from RNA extracted from *pOpON::CsEXPA1* etiolated hypocotyls
Data format	Raw (FASTQ) sequences
Experimental factors	RNA used for library preparation and sequencing was isolated from transgenic etiolated Arabidopsis hypocotyls with inducible suppression of targeted endogenous expansin genes on day 3 and day 5 after sowing
Experimental features	RNA-seq data was obtained from 3′mRNA sequencing to estimate gene abundance in count per million (CPM) represent the expression level of each transcripts
Data source location	Bangi, Malaysia (Transgenic seeds were obtained from Fleming lab, Department of Animal and Plant Sciences, University of Sheffield)
Data accessibility	http://www.ncbi.nlm.nih.gov/sra/SRP076440

**Direct link to deposited data**

http://www.ncbi.nlm.nih.gov/sra/SRP076440.

**Value of the data**

• This is the first transcriptome data of etiolated Arabidopsis hypocotyl overexpressing an expansin gene compared with previous reports [4], [5] on the suppression of endogenous expansin genes in Arabidopsis.
  The overexpression of *CsEXPA1* in tomato resulted in shorter plants [2], and altered cell wall composition in petunia flower petal [3].
• The present dataset is valuable for the identification of the genes which respond to expansin gene overexpression during the early and late phases of hypocotyl growth.
• This information will be useful for investigating global gene expression during hypocotyl growth, and for studying the effect of overexpressing a cell wall loosening factor on other cell wall related genes or regulatory factors to identify possible feedback mechanism responsible for growth defect.

## 1 Data

Data reported here describe the sequencing results (Table 1) obtained from the control and dex-treated *pOpON::CsEXPA1* Arabidopsis hypocotyls harvested on day 3 and day 5; each set with three biological replicates. This transcriptomic dataset was generated by QuantSeq. 3′ mRNA sequencing [6]. A total of twelve raw sequence data were deposited into NCBI SRA database and can be accessed with the BioProject accession number SRP076440 (http://www.ncbi.nlm.nih.gov/sra/SRP076440) under the BioSample accession number SAMN05192734.

**Table 1:** Statistics on *pOpON::CsEXPA1* Arabidopsis hypocotyl QuantSeq sequencing.

**Treatment**	**Day**	**Samples**	**Accession no.**	**Total reads**	**No. of reads mapped**	**% Reads mapped**
Control		Cso3_1	SRX2060156	5,794,866	4,255,162	73.4
	3	Cso3_21	SRX2060160	6,411,365	4,799,275	74.9
		Cso3_3	SRX2060157	6,061,856	4,750,661	78.4
		Cso5_13	SRX2060162	5,902,569	4,750,661	80.5
	5	Cso5_17	SRX2060163	6,434,928	3,937,326	61.2
		Cso5_9	SRX2060161	5,466,250	3,315,525	60.7
Dex-treated		Csx3_2	SRX2060164	5,709,604	4,379,551	76.7
	3	Csx3_22	SRX2060166	6,381,791	4,674,993	73.3
		Csx3_4	SRX2060165	5,528,108	4,214,720	76.2
		Csx5_10	SRX2060167	5,672,635	3,389,419	59.8
	5	Csx5_14	SRX2060158	7,315,534	4,336,244	59.3
		Csx5_18	SRX2060159	6,518,282	4,365,453	67.0

## 2 Experimental design, materials, and methods

### 2.1 Plant materials and treatments

This study utilised previously reported transgenic Arabidopsis line *pOpON::CsEXPA1* [1] containing a dex-inducible transactivating system, which allowed the induced ectopic overexpression of a heterologous cucumber expansin gene (*CsEXPA1*). Seed sowing, growing media and conditions followed as previously described [1], in the dark with petri dishes double wrapped in aluminium foil and placed vertically. For induction, growth media were supplemented with 10 µM of dexamethasone (dex). Control media were supplemented with an equivalent concentration of solvent DMSO (0.1% v/v). Etiolated hypocotyls samples were harvested on day 3 (early phase) and day 5 (late phase) after seed sowing. A total of 100 hypocotyls were pooled as one biological replicate. Three biological replicates were sampled for each treatment at each time point, totalling twelve samples.

### 2.2 RNA extraction, library construction and sequencing

RNA from pools of 100 hypocotyls was extracted using TRIzol (Invitrogen) according to manufacturer's instruction. RNA purity and integrity was measured using the ND-1000 Nanodrop spectrophotometer (Thermo Fisher Scientific Inc., USA) and Agilent 2100 bioanalyzer (Agilent Technologies, USA), respectively. RNA samples were cleaned using DNAse I kit according to the Rapid out removal DNA kit instruction (Thermoscientific) and converted into cDNA by using QuantSeq. 3′ mRNA-Seq Reverse (REV) Library Prep Kit (Lexogen) [6] according to manufacturer's instruction to generate compatible library for Illumina sequencing. cDNA libraries were assessed using TapeStation (Agilent Technologies, USA) before 100 bp single end sequencing using Illumina HiSeq. 2500 system at Australian Genome Research Facility (AGRF) based on standard protocols.

### 2.3 Transcriptome analysis

Raw sequencing reads (FASTQ) were processed individually to checked for per base sequence quality and screened for the presence of any Illumina adaptor/ overrepresented sequences and cross-species contamination through the AGRF quality control (QC) pipeline as per Lexogen QuantSeq data analysis workflow (https://www.lexogen.com/quantseq-data-analysis/). To quantify transcript abundance, the processed reads (FASTA) were mapped to Arabidopsis genome reference (TAIR10-release-30 ftp://ftp.ensemblgenomes.org/pub/plants/release-30/fasta/arabidopsis_thaliana/dna/). The mapping was performed using bowtie2 [7] with stringent “end-to-end” alignment and all other parameters were set to default values according to recommended data analysis workflow by Lexogen. The counts of reads mapping to each known gene were summarised in CPM (count per million) values using the TAIR10 gene annotation with the featureCounts utility of the subread package [8]. This transcript abundance dataset can be utilised to study the genome-wide changes in gene expression during etiolated hypocotyl development from day 3 to day 5, and to identify differentially expressed genes which are affected by the overexpression of a heterologous *CsEXPA1* gene from cucumber. Data from this study can be compared with previously reported expression data from the suppression of endogenous expansin genes [5].
